# Mr. Smyth, on Metallic Bougies

**Published:** 1807-09

**Authors:** Wm. Smyth

**Affiliations:** Tavistock Street


### *To the Editors of the Medical and Phyſical Journal.*

GENTLEMEN,

IN the communication I made to you in a late number of your useful work, page 460, on the improved state of the flexible metallic bougies and catheters, I mentioned I had carried them to every size which was considered by the faculty useful to their success in practice.

The great recommendation of the caustic, held out by its favourers, has been the quickness of the cure, or decrease of the stricture; but it is acknowledged, that this also is a matter of uncertainty, and it has required to be continued longer than the parts could bear, and even failed then completely of a cure, nay, that in many cases after submitting to much pain, only a partial cure has been effected, and that in a few weeks a reproduction of disease has occurred. Taking these facts as they stand, will any practitioner say, that the caustic either admits a *general application* in the cure of strictures, that it promises relief with certainty, or that its application can be trusted in any case as safe? On the contrary, it appears to be, in almost every instance, severe, painful, and incomplete in its operation. But to judge of it more fully, let us state next the progress of the Flexible Metallic Bougie.

In the construction of this instrument, No. 1, is formed so small, that there is hardly a stricture of the urethra so complete which it will not with proper caution and perseverance pass; experience is no doubt necessary here, as in all other operations, that the first attempts may be successful; and I can with confidence say, that by attending minutely to the state of the stricture, and adopting the size of the instrument to the degree of occlusion in the passage, I have never failed in introducing the bougie, and thus in the end of removing the disease. When the bougie is past, it is generally my rule to allow it to remain for half an hour, and what is remarkable and peculiar to the metallic bougie is, that during this time the patient suffers no uneasiness from is retention, which is so much the case with other bougies. Hence, I conceive that it really possesses a soothing or anodyne quality, connected certainly, as I have formerly stated, with galvanic principles. On the second application of the bougie, I begin with No. 1, as on the preceding day, to judge whether the advantage first gained over the stricture ture continues, and finding it pass readily I then introduce No. 2, which is retained for an equal length of time as the instrument at first, and by this regular daily succession of applications, and gradual enlargement of the size of the instrument, the disease comes to be completely removed in the space of two or three weeks at farthest, without any inconvenience to the patient, or any symptoms arising to prevent his ordinary pursuits, and with his acquiring rather better health while under the influence of the cure, as the discharge of the bladder goes on more regularly.

Some surgeons, while they decry the use of the caustic in the form employed by Mr. Hunter, have still not abandoned the principle, but have expected to get the better of its hurtful consequences by substituting another article of the same description, or the *kali purum*. This, instead of being an improvement, I should conceive, is still more to be dreaded than the lunar caustic, for in its action it is less circumscribed, and more apt to affect the surrounding parts, which is in fact, the leading objection to the use of caustics at all. What I contend against, is the general principle of cure, whatever the form of the caustic may be that is used; and since a method exists, as I have proved to the satisfaction of the most celebrated of the faculty, that is *easy, certain*, and attended with none of the dangerous effects which universally arise from the application of caustics of any description on such a delicate and irritable structure as the urethra; the employment of caustics, I flatter myself, will appear both a *useless* as well as *pernicious* practice. It is even the more so, since its greatest favourers admit, that in order to perfect any cure, the use of a bougie is still necessary. If, therefore, it is necessary in the progress, the same means will equally avail in the first instance, and be much more agreeable to the patient.

In closing my Remarks, I cannot here omit the paragraph of a letter from a patient in Edinburgh, who has just now applied to me, and had formerly submitted to the caustic for a cure, where it not only failed, but my correspondent observes, "that from the time he had *suffered* the operation of the caustic, the urethra had been constantly ulcerated;" a proof of the justice of my observations.

I am, &c.

WM. SMYTH.

*Tavistock Street*, 15 *June*, 1807.

